# Prevalence of hepatitis E virus in swine in China: a systematic review with meta-analysis (2004–2023)

**DOI:** 10.3389/fvets.2024.1472658

**Published:** 2025-02-27

**Authors:** Zhenwen He, Dingyu Liu, Baoling Liu, Pian Zhang, Xiaohu Wang, Gang Wang, Yuan Huang, Jing Chen, Rujian Cai

**Affiliations:** ^1^Key Laboratory of Livestock Disease Prevention of Guangdong Province, Scientific Observation and Key Laboratory for Prevention and Control of Avian Influenza and Other Major Poultry Diseases, Ministry of Agriculture and Rural Affairs, Institute of Animal Health, Guangdong Academy of Agricultural Sciences, Guangzhou, China; ^2^College of Animal Science and Technology, Zhongkai University of Agriculture and Engineering, Guangzhou, China; ^3^Dongguan Zhongtang Town Agricultural Technical Service Center, Dongguan, China; ^4^College of Veterinary Medicine, Huazhong Agricultural University, Wuhan, China

**Keywords:** hepatitis E virus, swine, China, prevalence, meta-analysis, systematic review, China swine hepatitis, epidemiological studies

## Abstract

**Introduction:**

Hepatitis E virus (HEV) is a major cause of acute hepatitis in humans and recognized as a zoonotic pathogen, with swine serving as a primary reservoir. Despite substantial research, comprehensive analysis encompassing regional variations and pig growth stages within China, as well as the influence of recent biosecurity measures on HEV prevalence, remains limited. In this study, we aim to assess the prevalence and risk factors associated with swine HEV in China.

**Methods:**

A thorough review of HEV infection studies was conducted using six databases: China National Knowledge Infrastructure, Wanfang, Wipro, Centre for Agriculture and Biosciences International, PubMed, and ScienceDirect, covering publications from January 1, 2004 to December 31, 2023. Eighty-seven studies investigating the seroprevalence of swine HEV IgG antibodies and HEV RNA detection rates were included. A rigorous meta-analysis and quality assessment followed.

**Results:**

The combined seroprevalence of swine HEV IgG antibodies was 58.0% (95% confidence interval [CI]: 52.0–65.0). The seroprevalence from 2019 to 2023 was lower (27.4, 95% CI: 26.3–28.2) than that in other years. The seroprevalence was higher in sows (67.2, 95% CI: 55.8–78.7) than in suckling, nursery, and fattening pigs. The detection rate of HEV RNA was 13.0% (95% CI: 11.0–15.0), with fattening pigs showing a significantly higher positivity rate (16.9, 95% CI: 13.2–20.7) than sows and suckling pigs. HEV RNA detection was significantly lower in bile (8.3, 95% CI: 6.3–10.3) than in feces and liver.

**Discussion:**

This study highlights the widespread presence of HEV in pig farms across China, with prevalence strongly linked to pig growth stage, study year, and sample type. The findings underscore the importance of pig growth stage, sample type, and recent biosecurity measures in controlling HEV prevalence, providing actionable insights for improving biosecurity practices in pig farms.

## 1 Introduction

Hepatitis E virus (HEV) is a major cause of acute hepatitis (1). It is a single-stranded positive-sense RNA virus from the *Hepeviridae* family, classified into eight genotypes (HEV-1 to HEV-8). HEV-1 and HEV-2 primarily infect humans, while HEV-3 and HEV-4 are found in various animals and are transmitted zoonotically (2). HEV infection is typically mild, presenting symptoms such as jaundice, malaise, fever, nausea, and vomiting, with most individuals recovering spontaneously within a few weeks. However, it can be more severe in pregnant women and immunocompromised individuals (3).

According to the World Health Organization, HEV represents a significant health risk, particularly in developing countries where poor sanitation and contaminated drinking water contribute to the virus spread (4). In recent years, an increasing number of indigenous HEV infections have been reported in developed countries, often linked to zoonotic transmission of HEV-3 and HEV-4 through the consumption of raw pig products and undercooked wild boar meat (5). HEV-3 and HEV-4 RNAs have been detected in the global pork supply chain, with pigs playing a key role in transmitting these genotypes to humans (6).

In China, where pork is a dietary staple, swine HEV poses a substantial public health risk, with seroprevalence among pigs ranging from 1.8 to 73% (7). Notably, outbreaks have been linked to the consumption of HEV-contaminated pig products (8). Another outbreak in Qingdao in July 2018 was also likely related to the consumption of HEV-contaminated pig livers (9). A study in Yunnan Province found that HEV gene sequences from 243 pig samples were highly homologous to human isolates (10), consistent with findings from other studies in China (11). Therefore, the prevalence of swine HEV in China poses a substantial threat to public health, necessitating ongoing monitoring of its risk to humans. Although many studies have examined swine HEV prevalence, a comprehensive synthesis is lacking. This study conducts a meta-analysis to evaluate swine HEV prevalence in China from 2004 to 2023, exploring factors influencing anti-HEV IgG and HEV RNA detection. This analysis aims to provide essential data and a theoretical basis for the prevention and control of swine HEV in China.

## 2 Methods

### 2.1 Study design

This review focuses on cross-sectional studies that investigated swine HEV prevalence in China. These studies were included because they provide a snapshot of HEV prevalence at a specific point in time, which aligns with the aim of this meta-analysis to summarize and evaluate the epidemiological data on swine HEV in China.

### 2.2 Participants

The studies included in this review focused on pigs as the study population, specifically examining the presence of HEV in swine populations across different regions of China.

### 2.3 Systematic review protocol

In this study, we followed the Preferred Reporting Items for Systematic Reviews and Meta-Analyses (PRISMA) guidelines (12) to search for relevant literature on swine HEV epidemiological investigations in China.

### 2.4 Data sources

We conducted searches across six databases: China National Knowledge Infrastructure, Wanfang, Wipro, Centre for Agriculture and Biosciences International (CABI), PubMed, and ScienceDirect. The search was limited to literature published from January 1, 2004, to December 31, 2023, in both Chinese and English. For English-language databases (CABI, PubMed, and ScienceDirect), we used the search terms: “swine,” “boar,” “pig,” “survey,” “prevalence,” “occurrence,” “Hepatitis E,” “virus,” “HEV,” and “China,” combined with Boolean operators “AND” and “OR.” In the Chinese databases, we used the keywords “pig,” “Hepatitis E” (Chinese abbreviation), “Hepatitis E” (Chinese full name), and “HEV.” The full search strategy is outlined in Table 1.

**Table 1:** Study search strategy.

Search platform	Search strategy	Outputs
CABI	[[All: swine] OR [All: boar] OR [All: pig]] AND [[All: survey] OR [All: prevalence] OR [All: occurrence]] AND [All: Hepatitis E] AND [[All: virus] OR [All: HEV]] AND [Geographic Locations: China]	33
PubMed	#1:“swine”[MeSH Terms] OR “swine”[All Fields] OR “swines”[All Fields] OR “pig”[All Fields] OR “boar”[All Fields]	228
	#2:“Hepatitis E”[MeSH Terms] OR “Hepatitis E”[All Fields] OR (“Hepatitis”[All Fields] AND “water”[All Fields] AND “borne”[All Fields]) OR “hepatitis, water borne”[All Fields]	
	#3:“China”[MeSH Terms] OR “China”[All Fields] OR “China’s”[All Fields] OR “Chinas”[All Fields]	
	#4 #1 AND #2 AND #3 AND (2004:2024[pdat])	
ScienceDirect	Year: 2004–2024	39
	Title, abstract, keywords: (swine OR boar OR pig) AND (hepatitis e virus OR HEV) AND (China)	
CNKI	(keywords: swine (fuzzy)AND (keywords: Hepatitis E + Hepatitis E virus + HEV(fuzzy))	368
Wan Fang Database	(All: (swine) and all: ((Hepatitis E OR Hepatitis E virus OR HEV)) and Publication time: 2004–2024	722
China Science and Technology Journal Database	(Title or keyword = swine AND (((((Title or keyword = hepatitis e OR Title or keyword = E Hepatitis) OR Title or keyword = Hepatitis E) OR Title or keyword = Viral Hepatitis E) OR Title or keyword = HEV) OR Title or keyword = Hepatitis E virus)) AND (years:[2004 to 2024])	825

We excluded duplicate review articles and screened the remaining studies according to the following inclusion criteria: [1] study subjects were pigs; [2] the study investigated swine HEV prevalence in China; [3] the study reported the number of pigs examined and the number that tested positive; [4] the sampling period was between 2004 and 2023; and [5] the study design was cross-sectional.

### 2.5 Study selection and data extraction

Data were extracted and cross-checked by two independent researchers. Any disagreements were resolved through discussion with the authors. The following information was extracted from eligible studies: first author, study year, geographic area, number of pigs at each growth stage, sample type, sampling site, total number of pigs tested, and number of positive cases. This data was compiled into a database using Microsoft Excel (version 2017).

The quality of the literature was assessed using a scoring system (13) based on five key questions, each scored 1 point: [1] Was the sample randomized in the study? [2] Were the sampling times accurately recorded? [3] Were the sampling locations representative? [4] Was the sample type described in detail? [5] Was the testing method accurately described? Papers were categorized as high-quality (4–5 points), medium-quality (2–3 points), or low-quality (1–2 points) based on their scores.

### 2.6 Research Indicator

The primary outcomes of interest were the prevalence rates of anti-HEV IgG antibodies and HEV RNA in pigs. These were defined as the proportion of positive samples relative to the total number of samples.

### 2.7 Data analysis

Meta-analyses were conducted separately to determine the detection rates of anti-HEV IgG antibodies and HEV RNA in pigs. If a study reported multiple prevalence data points (i.e., from different pig herds or sample types), each data point was treated as a separate record. The analyses were performed using Stata version 16.0 software. To approximate a normal distribution, the Freeman–Tukey double inverse chord transformation was applied (14). A random-effects model was employed to assess significant heterogeneity in the HEV positivity rate, with effect sizes expressed as 95% confidence intervals. Heterogeneity among studies was evaluated using I^2^ statistics. If I^2^ was greater than 50% and *p* < 0.05, indicating significant heterogeneity, a random-effects model was used. If I^2^ was less than 50% and *p* > 0.05, indicating less heterogeneity, a fixed-effects model was applied. Forest plots were generated for the overall meta-analysis, and funnel plots, along with Egger’s test, were used to assess publication bias. Sensitivity analyses were performed to evaluate the stability of the results. Subgroup and meta-regression analyses were conducted to identify sources of heterogeneity and analyze risk factors for HEV infection.

The risk factors examined included sampling area, pig growth stage, year of study, sampling site, sample type, and paper quality. Sampling areas were classified into seven regions based on administrative divisions in China: Northeast, North, East, South, Central, Northwest, and Southwest. Pig growth stages were classified as lactating pigs (<1 month of age), nursery pigs (1–3 months), fattening pigs (3–6 months), sows (>6 months), and breeding gilts (>6 months). The studies were grouped into three phases based on publication years: 2004–2010, 2011–2018, and 2019–2023. Sampling sites included large-scale farms, free-range farms, and slaughterhouses. Sample types included serum, feces, pig liver, and bile. Paper quality was categorized as high, medium, or low based on the literature quality scores.

## 3 Result

### 3.1 Literature search

**Figure 1:**
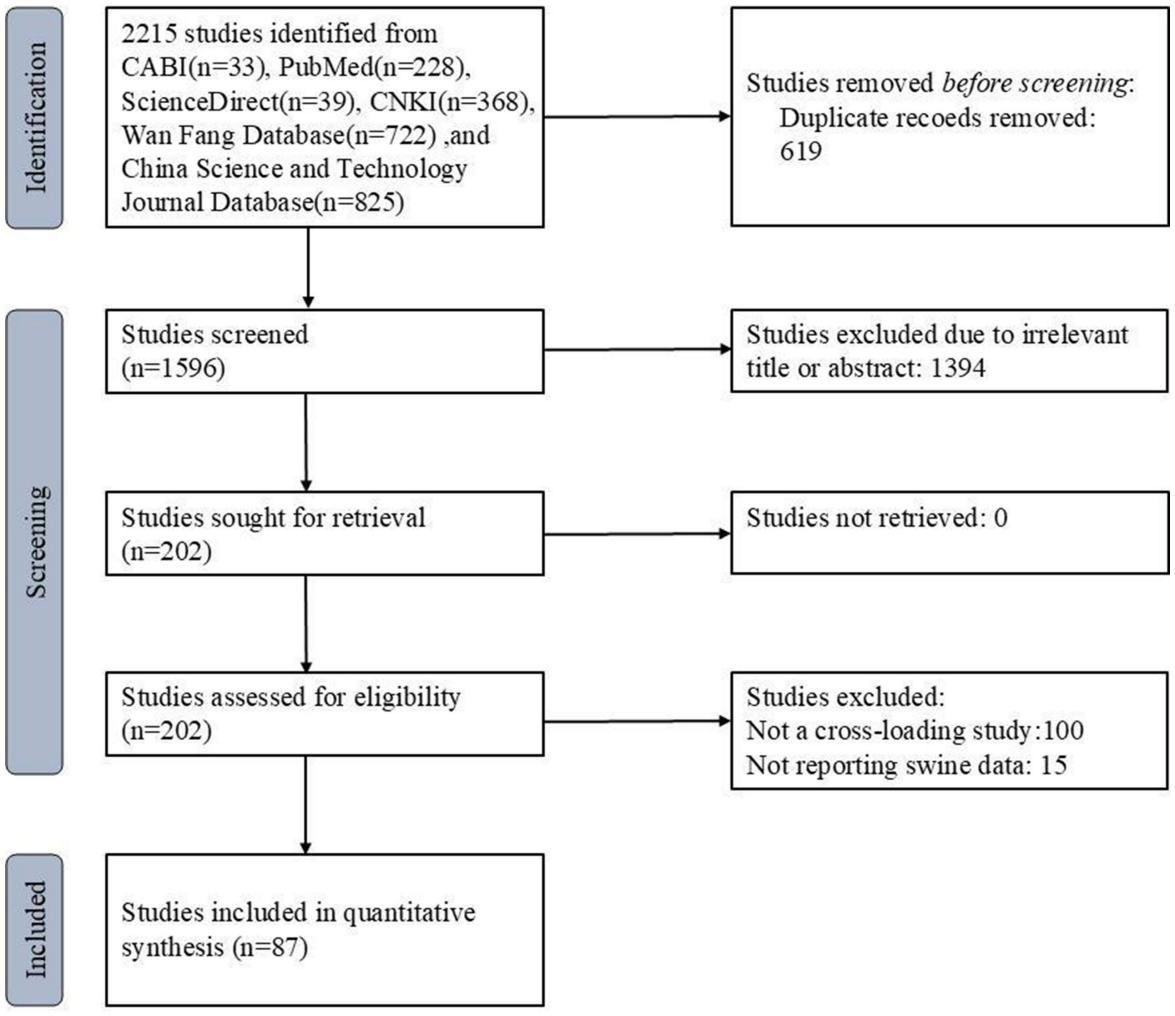
PRISMA flow diagram of the systematic review from the initial search and screening to the final selection of publications included in the study.

A total of 2,215 articles were retrieved from six databases. Figure 1 illustrates the study selection process, starting with the initial 2,215 articles retrieved from six databases, followed by screening for duplicates, titles, abstracts, and full texts. Ultimately, 87 studies were included in the meta-analysis. After removing duplicates and screening titles, abstracts, and full texts, 87 eligible studies were included in this meta-analysis. Among these, 68 studies were used to analyze the rate of anti-HEV IgG antibodies, and 27 studies were used to analyze the rate of HEV RNA in pigs. The selection process is shown in Figure 1. All studies used a cross-sectional design. Based on our quality criteria, 41 studies were classified as high quality (4–5 points), 45 as medium quality (2–3 points), and 11 as low quality (0–1 point). The risk of bias was assessed based on a standard quality assessment tool [e.g., the Newcastle-Ottawa Scale for cross-sectional studies]. Studies were categorized into high, medium, or low quality based on criteria such as randomization, sample representativeness, and the adequacy of reporting. Tables 2, 3 provide detailed information on the studies regarding anti-HEV IgG antibodies and HEV RNA detection rates in pigs, respectively, included in the analysis. A detailed summary of study characteristics, including geographical distribution, sample sizes, and demographic details of the swine populations, is presented in Tables 2, 3. For example, studies were conducted across diverse regions in China, including [list of regions], with sample sizes ranging from [X] to [Y].

**Table 2:** Information on the positive rate of serum antibodies to IgG in swine HEV included in the analysis

Study ID	Year	Number of studies	Positive number	Positive rate	Region	Area	Sample type	Detection method	Literature quality rating
Ai (40)	2018	510	121	23.73%	Yunan	Southwest	Serum	ELISA	4
Bi (41)	2008	1559	1183	75.88%	Heilongjiang	Northeast	Serum	ELISA	5
Chang (42)	2009	390	321	82.31%	Beijing	North	Serum	ELISA	3
Chen (43)	2021	256	65	25.39%	Shanghai	East	Serum	ELISA	2
Chen (44)	2010	1500	1264	84.27%	Hunan	Central	Serum	ELISA	5
Du (45)	2007	69	9	13.04%	Hainan	South	Serum	ELISA	1
Du (46)	2016	245	116	47.35%	Sichuan	Southwest	Serum	ELISA	2
Duan (47)	2022	307	173	56.35%	Yunnan	Southwest	Serum	ELISA	3
Feng (48)	2010	1109	981	88.46%	Gansu	Northwest	Serum	ELISA	5
Fu (49)	2010	321	66	84.62%	Xinjiang	Northwest	Serum	ELISA	1
Fu (50)	2005	151	62	41.06%	Heilongjiang	Northeast	Serum	ELISA	5
		208	118	56.73%	Liaoning				
		110	13	11.82%	Jilin	Northeast			
		127	57	44.88%	Guangxi	South			
		65	57	87.69%	Shanghai	East			
		42	11	26.19%	Inner Mongolia	Northwest			
		242	154	63.64%	Chongqing	Southwest			
		99	95	95.96%	Qinghai	Northwest			
		91	79	86.81%	Yunnan	Southwest			
		23	20	86.96%	Xinjiang	Northwest			
Geng (51)	2010	598	481	80.43%	Beijing	North	Serum	ELISA	4
Geng (52)	2019	115	104	90.43%	Hebei	Northeast	Serum	ELISA	2
Gong (53)	2018	610	162	26.56%	Tibet	Southwest	Serum	ELISA	4
Jinshan(54)	2010	356	186	52.25%	Inner Mongolia	Northwest	Serum	ELISA	3
Ju (55)	2007	647	445	68.78%	Shanghai	East	Serum	ELISA	4
Mou (56)	2008	553	357	64.56%	Shanghai	East	Serum	ELISA	4
Li (57)	2009	90	50	55.56%	Yunnan	Southwest	Serum	ELISA	1
Li (58)	2018	7187	1111	15.46%	Shaanxi	Northwest	Serum	ELISA	5
Li (29)	2012	174	108	62.07%	Beijing	North	Serum	ELISA	2
Li (59)	2012	197	42	21.32%	Yunnan	Southwest	Serum	ELISA	2
Li (60)	2011	1559	1183	75.88%	Heilongjiang	Northeast	Serum	ELISA	5
Li (33)	2019	1516	372	24.54%	Jilin	Northeast	Serum	ELISA	5
Li (61)	2009	638	505	79.15%	Beijing	North	Serum	ELISA	4
Li (30)	2011	960	760	79.17%	Yunnan	Southwest	Serum	ELISA	4
Li (62)	2008	623	574	92.13%	Henan	Central	Serum	ELISA	4
Li (63)	2008	904	617	68.25%	Hunan	Central	Serum	ELISA	4
Liang (64)	2014	561	183	32.62%	Guangdong	South	Serum	ELISA	4
Liang (34)	2019	758	186	24.54%	Jilin	Northeast	Serum	ELISA	4
Zhang (65)	2017	906	384	42.38%	Guangxi	South	Serum	ELISA	4
Liu (66)	2009	2511	1673	66.63%	Jilin	Northeast	Serum	ELISA	5
Liu (67)	2007	840	670	79.76%	Hubei	Central	Serum	ELISA	4
Liu (68)	2012	850	659	77.53%	Guangxi	South	Serum	ELISA	4
Lu (69)	2008	152	120	78.95%	-	Southwest	Serum	ELISA	2
		171	43	25.15%	-	North			
Ma (70)	2004	813	503	61.87%	Xinjiang	Northwest		ELISA	4
Pen (71)	2016	225	138	61.33%	Guangdong	South	Serum	ELISA	2
Qing (72)	2018	328	104	31.71%	Henan	Central	Serum	ELISA	3
Qing (73)	2010	537	448	83.43%	Guangxi	South	Serum	ELISA	4
Ren (74)	2009	434	354	81.57%	Guizhou	Southwest	Serum	ELISA	3
Ren (35)	2019	396	136	34.34%	-	Northeast	Serum	ELISA	3
Shao (75)	2009	820	597	72.80%	Jilin	Northeast	Serum	ELISA	4
Shi (76)	2013	421	204	48.46%	Tibet	Southwest	Serum	ELISA	3
Shuai (77)	2009	1330	740	55.64%	Zhejiang	East	Serum	ELISA	5
Tang (78)	2015	2880	1191	41.35%	Tibet	Southwest	Serum	ELISA	5
Tong (79)	2017	1127	779	69.12%	Guangdong	South	Serum	ELISA	5
Wang (80)	2007	190	176	92.63%	Hainan	South	Serum	ELISA	2
Wei (81)	2007	250	66	26.40%	Guangxi	South	Serum	ELISA	2
Wu (36)	2017	1660	884	53.25%	Xinjiang	Northwest	Serum	ELISA	4
Wu (82)	2023	599	415	69.28%	Xinjiang	Northwest	Serum	ELISA	4
Xie (83)	2008	430	372	86.51%	Jiangxi	East	Serum	ELISA	3
Yan (84)	2010	553	357	64.56%	Shanghai	East	Serum	ELISA	4
Yan (85)	2010	270	165	61.11%	Yunnan	Southwest	Serum	ELISA	2
Yang (86)	2011	450	105	23.33%	Jilin	Northeast	Serum	ELISA	3
Ye (87)	2009	159	106	66.67%	Fujian	East	Serum	ELISA	2
Zhai (88)	2022	226	144	63.72%	Henan	Central	Serum	ELISA	2
Zhang (89)	2010	80	56	70.00%	Hubei	Central	Serum	ELISA	1
Zhang (90)	2012	484	348	71.90%	Guangdong	South	Serum	ELISA	3
Zhang (31)	2017	174	66	37.93%	Hubei	Central	Serum	ELISA	2
Zhang (91)	2008	1154	622	53.90%	Zhejiang	East	Serum	ELISA	5
Zhang (92)	2008	788	528	67.01%	-	East	Serum	ELISA	4
Zhang (93)	2011	133	106	79.70%	Henan	Central	Serum	ELISA	2
Zhang (94)	2017	426	236	55.40%	Yunan	Southwest	Serum	ELISA	3
Zhang (95)	2020	1600	657	41.06%	Guizhou	Southwest	Serum	ELISA	5
Zhen (96)	2018	946	331	34.99%	Jilin	Northeast	Serum	ELISA	4
Zhou (97)	2006	417	299	71.70%	Shanghai	East	Serum	ELISA	3
Zhu (98)	2007	493	427	86.61%	Jilin	Northeast	Serum	ELISA	3
Zhu (99)	2006	1798	1600	88.99%	Shanghai	East	Serum	ELISA	5
Zuo (100)	2022	5096	940	18.45%	Yunnan	Southwest	Serum	ELISA	5

**Table 3:** Information on the detection rate of swine HEV RNA included in the analysis.

Study ID	Year	Number of studies	Positive number	Positive rate	Region	Study area	Sample type	Detection method	Literature quality rating
Ai (101)	2009	380	46	12.11%	Jiangsu	East	Bile	RT-nPCR	3
Cao (102)	2021	332	68	20.48%	Sichuan	Southwest	Feces	RT-nPCR	3
Chang (47)	2009	83	16	19.28%	Beijing	North	Feces	RT-nPCR	3
Chen (49)	2010	450	97	21.56%	Hunan	Central	Feces	RT-nPCR	5
Cheng (103)	2018	90	11	12.22%	Heilongjiang	Northeast	Liver	RT-nPCR	1
Fan (104)	2023	339	36	10.62%	Shangdong	East	Bile	RT-nPCR	3
		261	25	9.58%			Feces		
He (105)	2017	104	11	10.58%	Guangxi	South	Feces	RT-nPCR	2
Huang (106)	2012	78	8	10.26%	Yunnan	Southwest	Feces	RT-nPCR	1
Ji (107)	2017	73	5	6.85%	Guangdong	South	Bile	RT-nPCR	1
Gong (108)	2012	34	1	2.90%	Shangdong	East	Bile	RT-nPCR	1
Lei (60)	2008	61	10	16.39%	Shanghai	East	Feces	RT-nPCR	4
		334	35	10.48%	Sichuan	Southwest			
		65	15	23.08%	Shanghai	East			
		163	25	15.34%	Hebei	North			
		600	106	17.67%	Heilongjiang	Northeast			
		216	49	22.69%	Guizhou	Southwest			
		209	17	8.13%	Guizhou	Southwest			
		35	3	8.57%	Shanghai	East			
		129	13	10.08%	Guangxi	South			
Wu (36)	2017	100	11	11.00%	Xinjiang	Northwest	Feces	RT-nPCR	4
Xia (109)	2009	600	47	7.83%	-	East	Bile	RT-nPCR	4
Xie (110)	2009	80	17	21.25%	Jiangxi	East	Feces	RT-nPCR	1
Yan (111)	2015	166	21	12.65%	Yunnan	Southwest	Feces	RT-nPCR	2
Yan (112)	2016	1494	172	11.51%	Jiangsu	East	Feces	RT-nPCR	5
Yang (113)	2022	229	38	16.59%	Sichuan	Southwest	Feces	RT-nPCR	2
Yang (31)	2011	160	12	7.50%	Jilin	Northeast	Feces	RT-nPCR	3
Zhang (89)	2010	135	21	15.56%	Hubei	Central	Liver	RT-nPCR	1
Zhang (93)	2011	200	15	7.50%	Henan	Central	Feces	RT-nPCR	2
Zhang (114)	2005	160	11	6.88%	Zhejiang	East	Bile	RT-nPCR	2
		132	13	9.85%			Feces		
Zhen (115)	2009	493	111	22.52%	Shanghai	East	Feces	RT-nPCR	3
Zheng (116)	2006	282	27	9.57%		East	Feces	RT-nPCR	2
Zhou (117)	2018	377	88	23.34%	Gansu	Northwest	Feces	RT-nPCR	3
Zhou (118)	2022	180	28	15.56%	Shanghai	East	Feces	RT-nPCR	2
Zhou (119)	2009	127	9	7.09%	Shanghai	East	Bile	RT-nPCR	5
		1699	191	11.24%			Feces		
Zhou (120)	2019	229	38	16.59%	Sichuan	Southwest	Feces	RT-nPCR	2

### 3.2 Publication Bias

**Figure 2:**
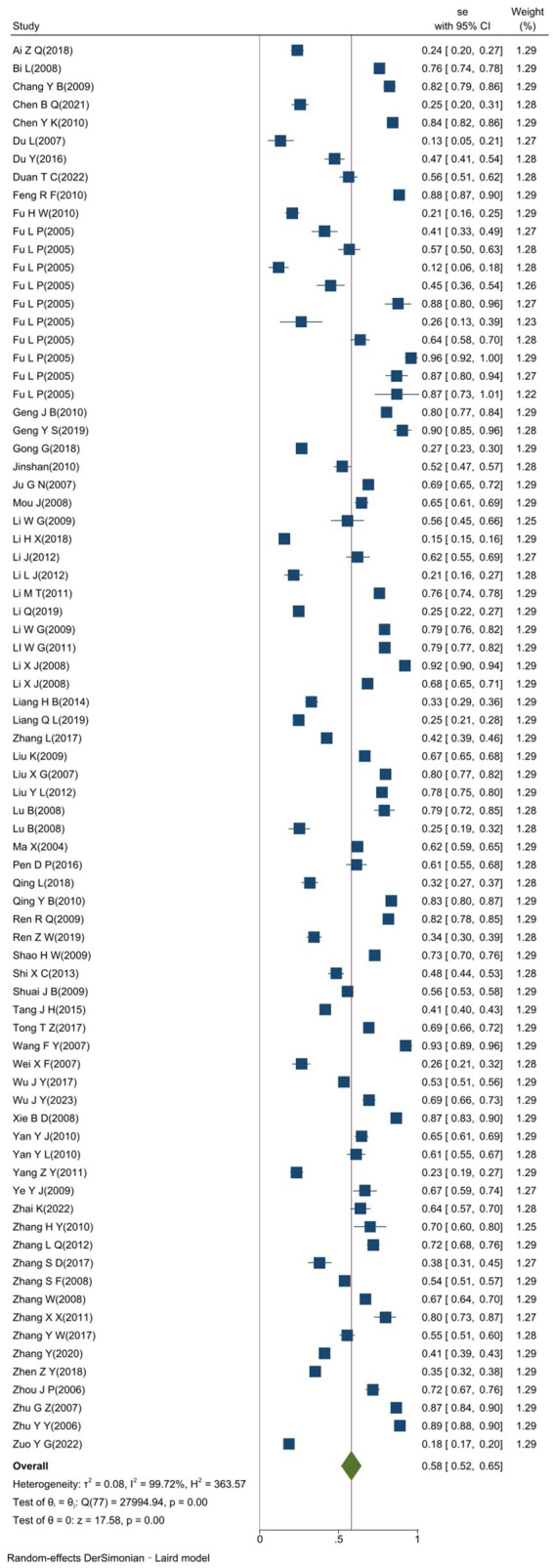
Forest plot of serum antibody IgG positivity rate of swine hepatitis E virus in studies conducted in China.

**Figure 3:**
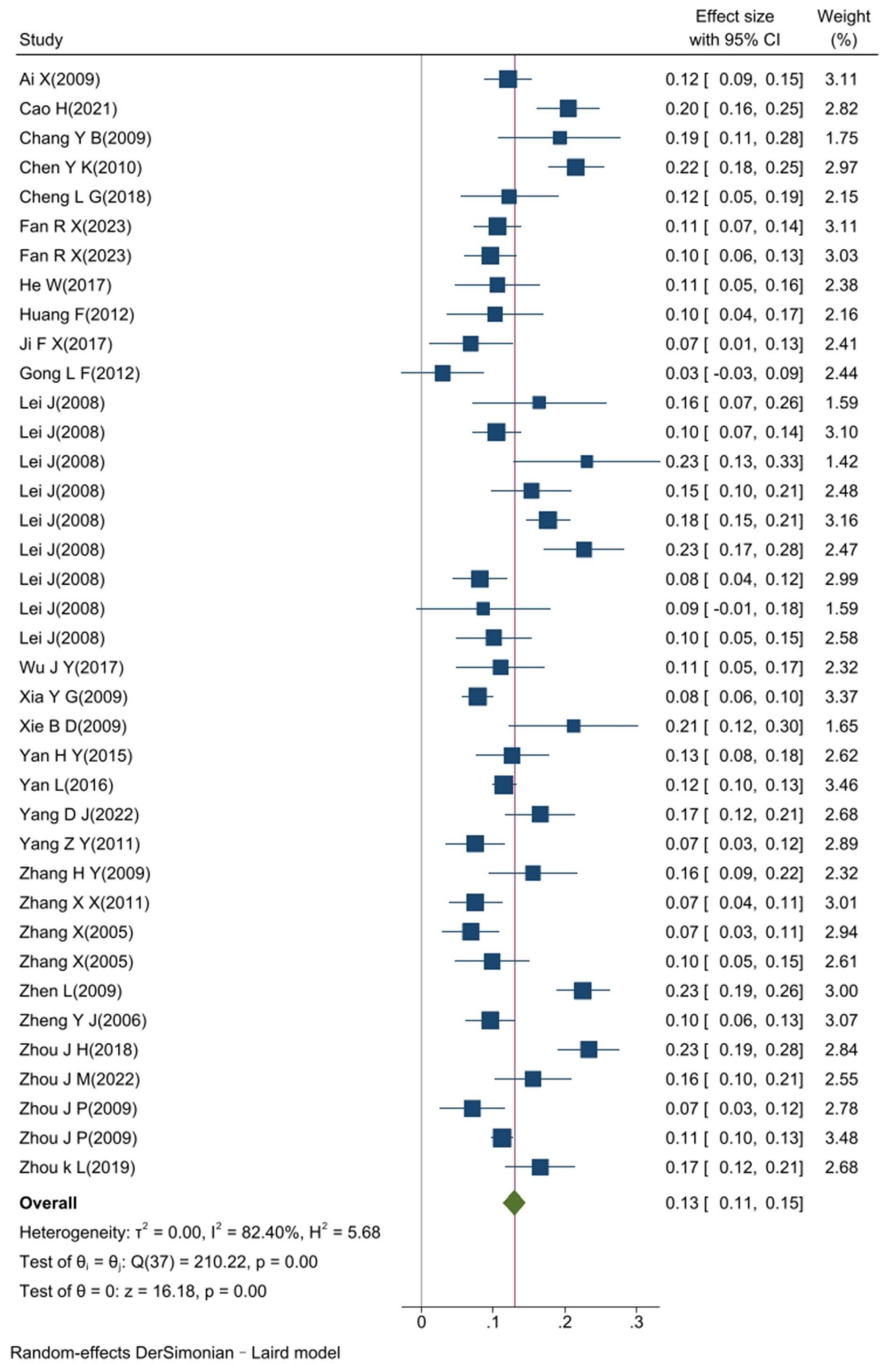
Forest plot of the RNA detection rate of swine hepatitis E virus in a study conducted in China.

**Figure 4:**
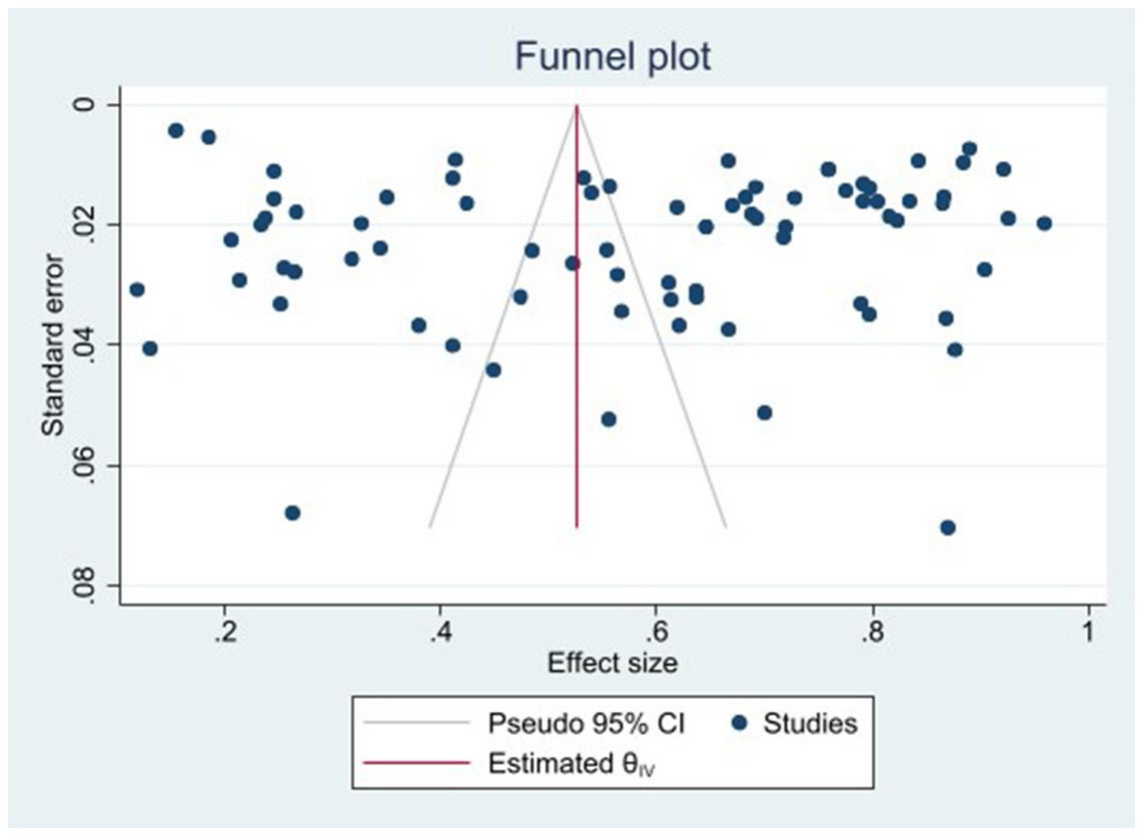
Funnel plot of serum antibody IgG positivity rate of swine hepatitis E virus in the literature.

**Figure 5:**
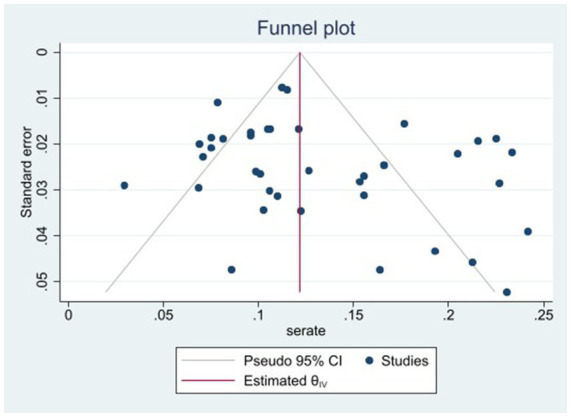
Funnel plot of swine HEV RNA detection rate in the literature.

**Figure 6:**
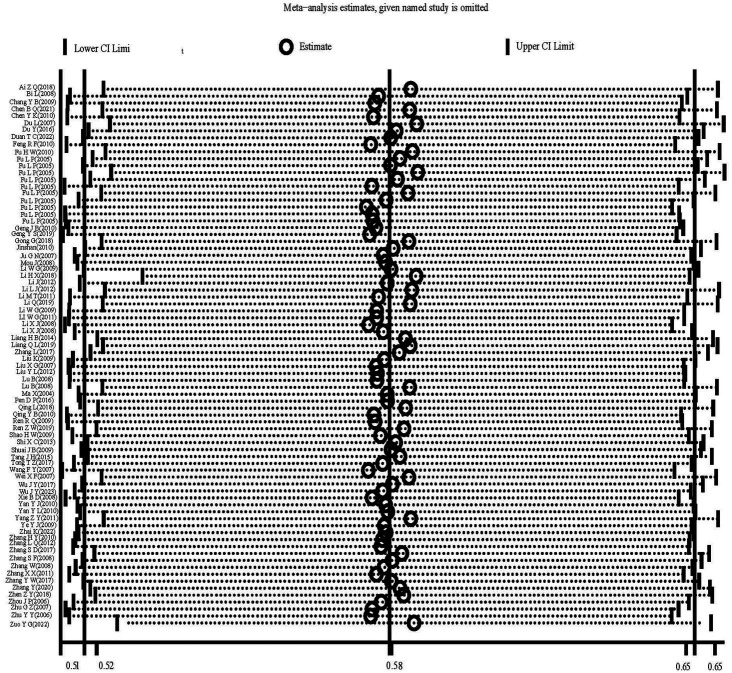
Sensitivity analysis of serum antibody IgG positivity rate of swine hepatitis E virus in the literature.

**Figure 7:**
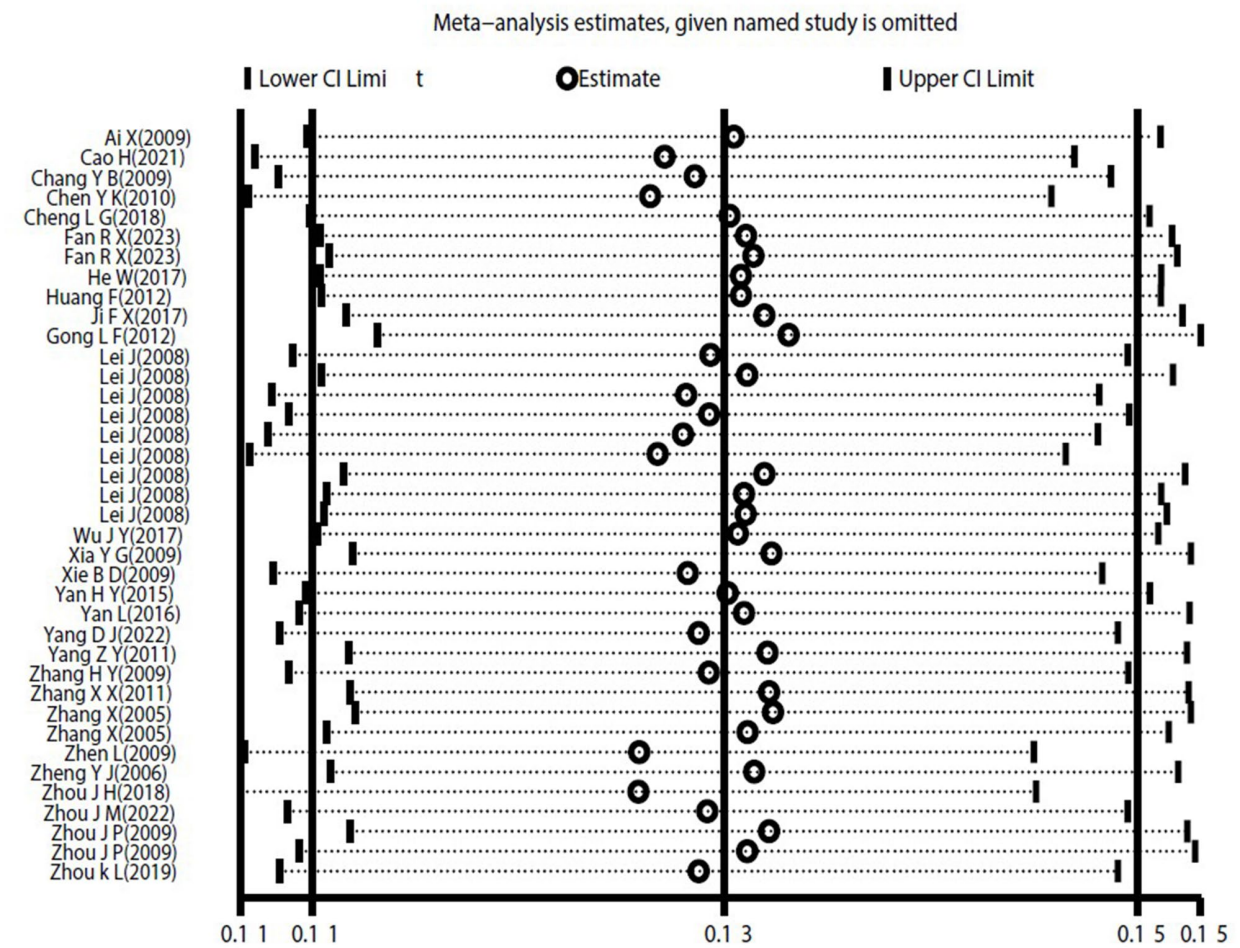
Sensitivity analysis o of swine HEV RNA detection rate in the literature.

The heterogeneity test results for studies analyzing anti-HEV IgG antibodies were I^2^ =99.72% and *p* < 0.001. For studies analyzing the RNA detection rate of swine HEV, the heterogeneity test results were I^2^ = 82.70%, *p* < 0.001, indicating significant heterogeneity in the selected studies. Therefore, a random-effects model was applied for the meta-analysis (Figures 2, 3). Funnel plot analysis revealed that the studies included in the analysis (represented as dots) had a largely symmetrical distribution within the funnel plot, indicating no significant publication bias (Figures 4, 5). Egger’s test results for anti-HEV IgG antibodies were *p* = 0.5527 (*p* > 0.05), and for HEV RNA detection rates, the *p*-value was 0.0572 (*p* > 0.05), indicating no significant publication bias. Sensitivity analysis showed that the combined effect sizes remained within the 95% CI when each study was excluded individually, demonstrating the stability and reliability of the meta-analysis results (Figures 6, 7).

### 3.3 Meta-analysis

#### 3.3.1 Overall analysis

**Figure 8:**
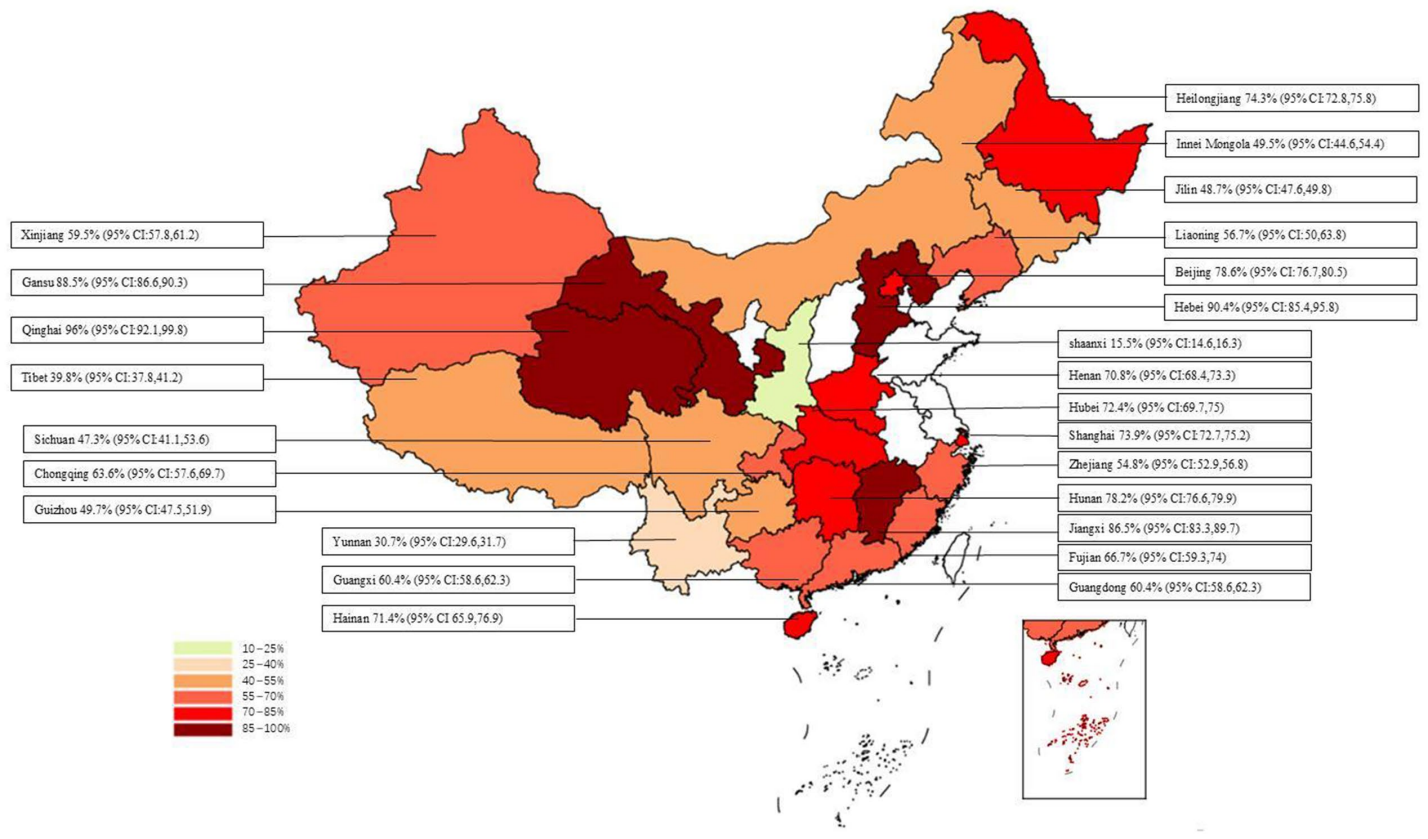
Positive rate of serum antibodies to IgG to swine HEV in different provinces/cities in China.

The overall positive rate for anti-HEV IgG antibodies was 58.0% (95% CI: 52.0, 65.0). No significant difference was observed between different provinces or cities (*p* = 0.612), as shown in Figure 8. The highest positive rate was observed in Qinghai Province at 96.0% (95% CI: 92.1, 99.8), followed by Hebei Province at 90.4% (95% CI: 85.4, 95.8), and Gansu Province at 88.5% (95% CI: 86.6, 90.3). Lower positive rates were found in Yunnan Province at 30.7% (95% CI: 29.6, 31.7) and Shaanxi Province at 15.5% (95% CI: 14.6, 16.3). The synthesized findings suggest substantial regional variation in the prevalence of anti-HEV IgG antibodies, with higher rates observed in [specific provinces]. These differences could reflect regional differences in farming practices, environmental factors, or pig management systems. No significant differences were found across different study years, indicating that the HEV prevalence rates have remained relatively stable over the past two decades.

**Figure 9:**
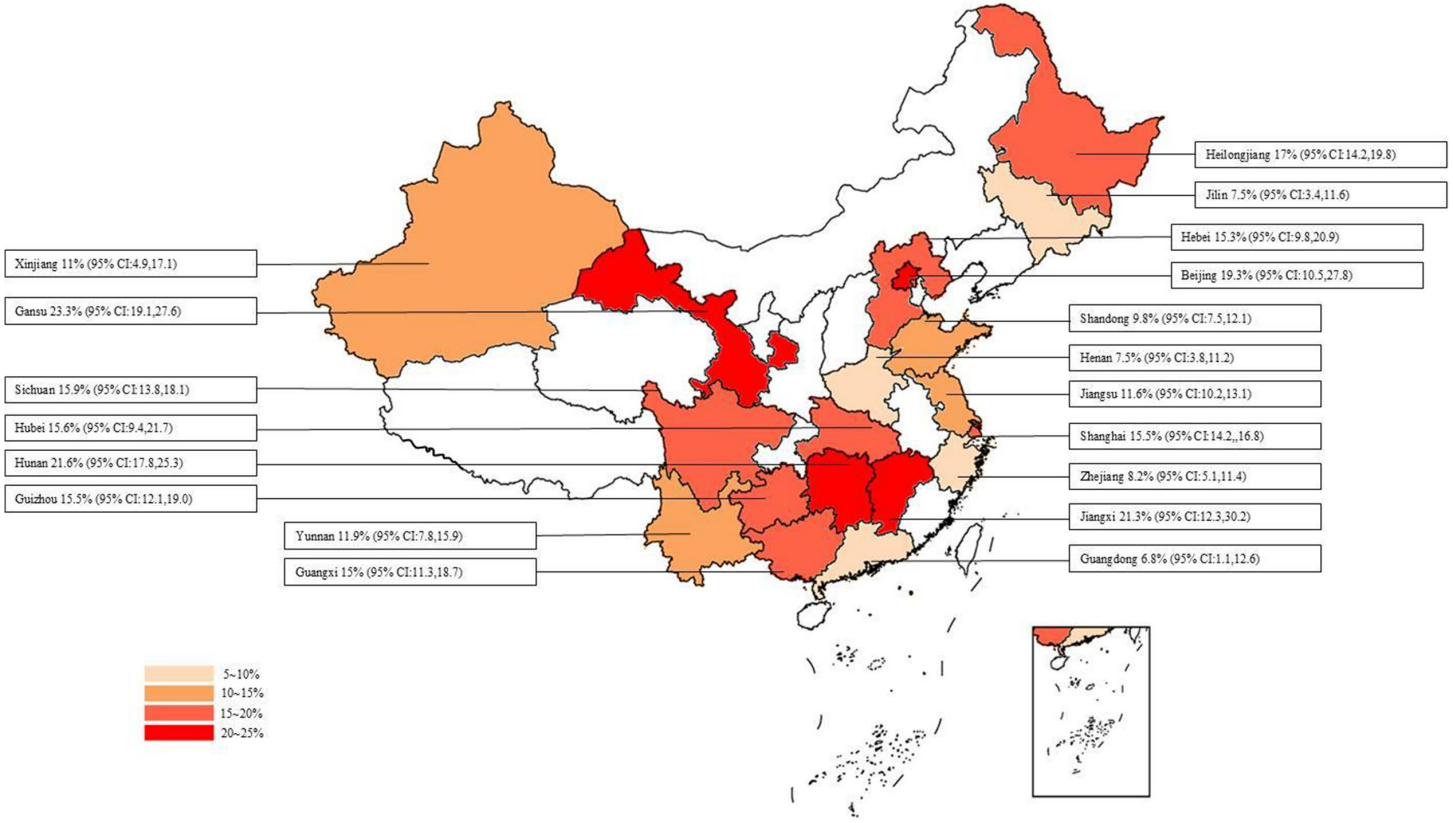
Swine HEV RNA positivity rates in different provinces/cities in China.

The overall detection rate of swine HEV RNA was 13.0% (95% CI: 11.0, 15.0), and no significant difference was observed among different provinces or cities (*p* = 0.120), as shown in Figure 9. The highest detection rate was observed in Gansu Province at 23.3% (95% CI: 19.1, 27.6), followed by Hunan Province at 21.6% (95% CI: 17.8, 25.3), and Jiangxi Province at 21.3% (95% CI: 12.3, 30.2). Lower detection rates were found in Henan Province at 7.5% (95% CI: 3.8, 11.2), Jilin Province at 7.5% (95% CI: 3.4,11.6), and Guangdong Province at 6.8% (95% CI: 1.1, 12.6).

#### 3.3.2 Subgroup analysis

The sources of heterogeneity in the included studies on anti-HEV IgG antibodies were analyzed across five subgroups: sampling area, pig growth stage, sampling site, sampling year, and literature quality (Table 4). The results showed significant differences in positivity rates among different growth stages (*p* = 0.009, *p* < 0.05). Sows had the highest positivity rate at 67.2% (95% CI: 55.8, 78.7), followed by fattening pigs at 59.5% (95% CI: 52.6, 66.4). Nursery pigs had a positivity rate of 49.5% (95% CI: 41.2, 57.8), breeding gilts had a rate of 45.0% (95% CI: 27.9, 62.1), and lactating pigs had the lowest rate at 34.3% (95% CI: 22.8, 45.8). Significant differences were observed between study years (*p* < 0.05). The highest positivity rate was found in studies from 2004 to 2010 at 72.0% (95% CI: 71.5, 72.6), followed by 40.5% (95% CI: 39.6, 41.2) in studies from 2011 to 2018, and the lowest rate was 27.4% (95% CI: 26.3, 28.2) in studies from 2019 onward. The sampling area, sampling site, and literature quality did not significantly contribute to the differences in anti-HEV IgG antibody rates (*p* > 0.05).

**Table 4:** Correlation analysis of risk factors for swine HEV antibody IgG prevalence in China.

Risk	No.	No. Examined	No.	rate(CI:95%)		Heterogeneity				Meta-regression				
factors	Studies		Positive			Q	P-value	I^2^	H^2^	P-value	Coefficient		R^2^/%	I^2^-ras/%
Area										0.996	0.00 (−0.003, 0.029)		7.60	99.67
Northeast	12	11592	6490	51.40%	(38.2,64.6)	3439.09	0.000	99.62	264.55					
North	5	1971	1458	66.10%	(50.7,81.4)	270.33	0.000	98.52	67.58					
East	12	8150	5548	66.80%	(56.6,76.9)	1242.14	0.000	99.11	112.92					
South	11	5326	3247	56.10%	(42.8,69.4)	1276.07	0.000	99.22	127.61					
Central	9	4808	3601	67.70%	(56.8,78.6)	717.18	0.000	98.88	89.65					
Northwest	8	11966	4272	63.40%	(38.7, 88.2)	7199.70	0.000	99.87	799.67					
Southwest	16	12163	5179	52.60%	(41.5, 63.8)	2918.58	0.000	99.45	182.41					
Growth stage										0.009	0.037 (0.009,0.064)		10.49	99.07
Lactating pigs	9	886	318	34.30%	(22.8,45.8)	100.58	0.000	92.05	12.57					
Nursery pigs	37	12039	6560	49.50%	(41.2,57.8)	3848.94	0.060	99.06	106.92					
Fattening pigs	42	11785	7614	59.50%	(52.6,66.4)	4083.17	0.000	99.00	99.59					
Sow	25	5735	3274	67.20%	(55.8,78.7)	2996.40	0.000	99.20	124.85					
Breeding gilts	10	1891	668	45.00%	(27.9,62.1)	598.56	0.000	98.50	66.51					
Location										0.444	−0.041 (−0.147, 0.064)		0.80	99.73
Farms	47	41198	22566	60.70%	(52.1,69.3)	21422.78	0.000	99.79	465.71					
Free-range farmers	9	2666	851	47.70%	(33.8,61.6)	392.11	0.000	97.96	49.01					
Abattoir	8	1666	745	56.10%	(35.0,77.1)	746.72	0.000	99.06	106.67					
Year of study										0.000	−0.150 (−0.218,-0.081)		49.50	99.48
2004~2010	35	24822	17877	72.00%	(71.5,72.6)	2703.53	0.000	98.71	77.24					
2011~2018	23	24613	9978	40.50%	(39.6,41.2)	7246.02	0.000	99.68	315.04					
2019~2023	10	9269	2535	27.40%	(26.4,28.2)	1540.60	0.000	99.48	192.58					
Quality of literature										0.412	0.026 (−0.035,0.086)		0.00	99.73
Low	4	560	181	39.50%	(15.3,63.7)	119.20	0.059	97.48	39.73					
Middle	27	8269	4830	58.00%	(49.0,66.9)	2591.23	0.058	98.96	95.97					
High	37	49758	25129	58.10%	(51.6,64.6)	24528.31	0.088	99.82	545.07					

*P<0.05 represents a significant difference.*

The studies on swine HEV RNA detection rates were analyzed using six subgroups: sampling area, pig growth stage, sample type, sampling site, sampling year, and literature quality (Table 5). The results were consistent with those for anti-HEV IgG antibodies, with pig growth stage being a significant factor (*p* = 0.006, *p* < 0.05). The highest detection rate of HEV RNA was 16.9% (95% CI: 13.2, 20.7) in fattening pigs, followed by 15.7% (95% CI: 11.9, 19.6) in nursery pigs. The detection rate of HEV RNA in sows and lactating pigs was significantly lower, at 8.4% (95% CI: 4.2, 12.3) and 6.9% (95% CI: 4.1, 9.7), respectively. Significant differences in HEV RNA detection rates were observed between sample types, with lower detection rates found in bile (8.3%) than in feces (14.6%) and liver (13.0%). The sampling area, sampling site, study year, and literature quality did not significantly influence the differences in HEV RNA detection rates (*p* > 0.05). Additionally, the analysis revealed that fattening pigs had the highest detection rate, likely reflecting their higher exposure to environmental risk factors.

**Table 5:** Correlation analysis of risk factors for molecular prevalence of swine HEV in China.

Risk	No.	No. Examined	No. Positive	rate(CI:95%)		Heterogeneity				Meta-regression			
factors	Studies					Q	P-value	I^2^	H^2^	P-value	Coefficient	R^2^/%	I^2^-ras/%
Area										0.283	0.004 (-0.003,0.011)	1.45	82.33
Central	3	785	133	14.80%	(5.5,24.2)	27.49	0.000	92.72	13.74				
East	12	6422	762	11.40%	(9.5,13.4)	77.95	0.000	79.47	4.84				
North	2	246	41	16.50%	(11.9,21.1)	0.58	0.450	0.00	1.00				
Northeast	3	850	129	12.60%	(5.5,19.6)	15.53	0.000	87.12	7.76				
Northwest	2	477	99	17.40%	(5.3,29.5)	10.48	0.000	90.46	10.48				
South	3	426	58	12.50%	(6.1,18.9)	13.36	0.000	77.55	4.45				
Southwest	6	1793	274	14.60%	(11,18.3)	35.56	0.000	80.32	5.08				
Growth stage										0.006	-0.032 (-0.054,-0.009)	11.43	86.76
Lactating pigs	4	630	48	6.90%	(4.1,9.7)	4.95	0.000	39.45	1.65				
Nursery pigs	12	2531	365	15.70%	(11.9,19.6)	91.18	0.000	85.74	7.01				
Fattening pigs	14	2341	426	16.90%	(13.2,20.7)	79.59	0.000	82.41	5.69				
Sow	8	1127	107	8.40%	(4.2,12.3)	50.40	0.000	86.11	7.20				
Location										0.220	-0.017 (-0.044,0.010)	0.00	80.57
Farms	23	7581	1095	14.70%	(12.7,16.8)	138.87	0.000	83.44	6.04				
Free-range farmers	3	330	50	13.80%	(12.1,15.5)	10.28	0.010	80.54	5.14				
Abattoir	5	1265	132	10.10%	(8.5,11.8)	4.69	0.450	0.00	1.00				
Sample type										0.015	-0.033 (-0.059,-0.006)	9.27	80.98
Feces	29	9026	1306	14.60%	(12.8,16.5)	167.71	0.000	83.30	5.99				
Bile	7	1713	155	8.30%	(6.3,10.3)	11.65	0.070	48.51	1.94				
Liver	2	260	35	13.00%	(8.9,17.1)	1.60	0.450	0.00	1.00				
Year of study										0.928	0.001 (-0.020,0.022)	0.00	82.83
2005~2010	11	6433	879	13.60%	(11.4,15.9)	132.84	0.000	84.40	6.64				
2011~2018	11	2876	355	10.70%	(7.7,13.6)	49.10	0.000	79.64	4.91				
2019~2023	5	1570	233	14.70%	(11.1,18.3)	21.08	0.001	76.28	4.22				
Quality of literature										0.580	0.008 (-0.022,0.036)	0.00	85.87
Low	6	490	63	11.00%	(6.1,15.9)	16.69	0.005	70.04	3.34				
Middle	15	6282	604	14.00%	(11.2,16.8)	101.39	0.000	86.19	7.24				
High	6	4107	800	13.10%	(11.4,14.9)	54.40	0.000	90.82	10.89				

*P<0.05 represents a significant difference.*

## 4 Discussion

In this study, we found that pig growth stage significantly influenced both the positivity rates of anti-HEV IgG antibodies and detection rates of HEV RNA in pigs, consistent with the findings of previous studies (15–17). As pigs enter the nursery stage approximately 1 month of age, maternal antibodies disappear, increasing their susceptibility to HEV and subsequently raising infection rates. This transition leads to a noticeable increase in both anti-HEV IgG antibody positivity and HEV RNA detection rates. In our study, anti-HEV IgG antibody positivity increased from 34.3% (95% CI:22.8,45.8) in lactating pigs to 49.5% (95% CI: 41.2, 57.8) in nursery pigs. Similarly, HEV RNA detection rates increased from 6.9% (95% CI: 4.1, 9.7) to 15.7% (95% CI: 11.9, 19.6). Serum IgG antibodies can persist for extended periods, which explains the higher positivity rates in older pigs. The duration of HEV viremia in pigs is estimated to be 2–3 weeks, after which the virus is gradually cleared following the production of serum antibodies. Consequently, pigs older than 6 months have typically experienced infection and cleared the HEV virus from their bodies (18). In this study, the HEV RNA detection rate reached a maximum of 16.9% (95% CI: 13.2, 20.7) at the fattening stage, whereas it decreased to a minimum of 8.4% (95% CL: 4.2, 12.5) in sows older than 6 months, which is consistent with previously reported results. The influence of the pig growth stage on HEV seropositivity was significant, with the highest positivity rates observed in pigs at the nursery stage. This aligns with the results of previous studies that showed increased vulnerability to HEV infection as maternal antibodies wane (15–17). Similarly, biosecurity measures introduced post-2019 appear to have substantially reduced the overall HEV prevalence in pig populations. These findings suggest that the combination of the growth stage transition and enhanced biosecurity practices plays a critical role in determining the HEV infection rates across different stages of pig development.

The global prevalence of IgG antibodies against porcine HEV varies between 20 and 100% (19). In Europe and the United States, most pig infections are attributed to HEV genotype 3, with reported HEV antibody positivity rates ranging from 40 to 88% in some countries (2). For instance, in Italy, the HEV antibody positivity rate in slaughtered pigs is 76.8% (20); in Bulgaria, it ranges from 40.0 to 60.3% (21); in Corsica, France, the rate in free-range pigs is 88% (22); and in the United States, the national average HEV antibody positivity rate in pigs is 40% (23). In comparison, our analysis suggests that the HEV antibody positivity rates in pigs in Europe and the United States tend to be higher than those observed in China. In Asia, HEV genotype 4 predominates, with HEV antibody positivity rates ranging from 35 to 73% (2). In Mongolia, the HEV antibody positivity rate in pigs is 35.5% (24), similar to the findings from China; in Japan, the rate in wild boars is between 5.0 and 15.3% (25), whereas in South Korea, the overall prevalence of anti-HEV antibodies in pigs is 14.8% (26), both of which are lower than those reported in China. By contrast, in Vietnam, the HEV antibody positivity rate in pigs is 58.5% (27), and in India, it is 65.0% (28), both higher than those observed in China. Overall, the HEV antibody positivity rate in pigs in China appears to be moderate when compared to that in other countries in Asia.

Epidemiological studies on swine HEV across various regions have yielded inconsistent results. For instance, Li et al. (29) tested pig sera from the suburbs of Beijing and found an anti-HEV IgG antibody positivity rate of 62.1%. Similarly, Li et al. (30) tested pig sera from farms in Yunnan Province and reported a positivity rate of 79.1%. In contrast, Zhang et al. (31) conducted testing on sera from pig farms in Anlu City, Hubei Province, and found a lower positivity rate of 38.5%. Despite differences in study methodologies, most reports suggest that the HEV antibody positivity rate in pigs across China is moderate, with variation primarily linked to genotype differences rather than geographical factors. This is consistent with our study’s findings, which showed no significant geographic variation in HEV prevalence despite regional differences in climate and farming practices. However, our study did not observe significant differences in the serum anti-HEV IgG antibody positivity or HEV RNA detection rates across pigs from different regions. This suggests that geographic factors are not significant determinants of HEV prevalence in the pig population. While geographic and climatic differences are often considered potential contributors to variations in HEV prevalence, they do not appear to be the main factors in this case. Instead, differences in the genotypes of HEV strains prevalent in each region are likely more influential (32). In this study, all HEV genotypes identified in pigs were genotype 4, with subtype 4a being the predominant form. Some studies also detected subtypes 4b and 4d. The consistent presence of similar HEV genotypes across regions may help explain why our study did not find significant geographic variation in HEV prevalence.

At the end of the fattening stage, approximately 6 months of age, pigs are typically sent to the slaughterhouse. It is reasonable to expect that the anti-HEV IgG antibody positivity rate in slaughterhouse pigs would align with that observed in pigs at the fattening stage on the farm, which our study confirmed. As indicated in our earlier analysis, pigs older than 6 months have generally experienced HEV infection, and the virus has been cleared from their bodies. This suggests that the likelihood of slaughterhouse pigs carrying HEV is relatively low. However, despite the significantly lower detection rate of HEV RNA in slaughterhouse pigs than in pigs on farms and in free-range settings, a detection rate of 10.1% (95% CI: 8.5, 11.8) still indicates the possibility of HEV-positive pigs entering slaughterhouses. Given that slaughterhouses are the initial point in pork production, HEV carried by these pigs could potentially lead to human infections via a foodborne route. Therefore, ongoing surveillance of HEV in slaughterhouse pigs is crucial to assess the risk of HEV transmission through pork products. Additionally, the presence of HEV in slaughterhouse pigs and wild boar populations highlights a significant public health risk, particularly through the consumption of undercooked pork and wild boar meat. Therefore, public health initiatives must focus on education about safe cooking practices and regulations on meat handling to prevent zoonotic transmission.

In this study, the HEV RNA detection rate in pig bile was significantly lower than that in pig liver and feces, which differs from the findings in some previous studies. This discrepancy may be attributed to the fact that the bile samples included in our analysis were exclusively sourced from slaughterhouses, whereas most fecal samples were obtained from pig farms. Previous analyses have shown that the HEV RNA detection rate in pigs from slaughterhouses is significantly lower than that in pigs from farms, which could explain why the RNA positivity rate in bile is even lower than that in fecal samples. In our analysis, fecal samples exhibited the highest HEV RNA detection rate, suggesting that fecal shedding plays a critical role in HEV transmission within pig populations. This supports the hypothesis that HEV spreads through the fecal–oral route, as contaminated feces facilitate environmental dissemination and subsequent transmission among pigs. Based on these findings, we recommend that pig farms prioritize manure management and environmental disinfection to interrupt the fecal-oral transmission of HEV within pig populations. Additionally, the HEV RNA detection rate in pig liver was 13.0%, underscoring the high risk of HEV carriage in this organ. This finding highlights the significant association between the consumption of undercooked liver and human HEV infection. To address this risk, monitoring programs should be developed across different regions to assess infection risks, and public awareness campaigns should be intensified to educate the public on the dangers of consuming undercooked liver and other raw pork products.

We categorized the included studies by research year and found a significant decline in the IgG antibody positivity rate in pig serum after 2019. African swine fever (ASF) began to emerge in China in 2018, drawing significant attention from both the industry and government. In 2019, the General Office of the State Council issued the “Opinions on Strengthening the Prevention and Control of ASF,” mandating pig farms to strengthen cleaning and disinfection, manage personnel and vehicle access, and prohibit feeding pigs with food waste. Furthermore, live pig transportation across the country was progressively restricted, and stringent measures were implemented to combat meat product smuggling. These actions were crucial in preventing the spread of ASF and also had a significant impact on the transmission of other pathogens. Consequently, we believe the enhanced biosecurity measures implemented in pig farms after 2019 reduced the risk of HEV transmission from the environment. Strengthening personnel and vehicle management, along with limiting live pig movement, helped reduce the risk of introducing external HEV strains, thereby significantly lowering HEV prevalence in pig populations. This underscores the importance of biosecurity measures—such as environmental disinfection, control of personnel, vehicles, and materials, and prohibition of food waste use in feeding pigs—in reducing HEV infection risk in pig populations.

In the studies included in our analysis, the majority focused on domestic pigs, with only four studies (33–36) reporting data on serum IgG antibody detection in wild boars, which showed a positivity rate of 38.0% (95% CI: 14.0, 63.0). Although the serum IgG antibody positivity rate in wild boars is lower than that in domestic pigs, wild boars play a crucial role in the transmission of HEV (37). Thus, the prevalence of HEV in wild boars in China should not be overlooked. Several studies have identified risk factors for human HEV infection, suggesting that the virus is transmitted through the consumption of wild boar meat. A case–control study in Germany found that a significant number of local HEV cases were linked to the consumption of undercooked wild boar meat and offal (38). Serological studies have also highlighted that direct contact with infected wild boars increases the risk of HEV infection (39). Consequently, it is essential for regions to monitor wild boar populations and manage their disease risks effectively to prevent zoonotic transmission of HEV to humans or livestock. In Japan, the implementation of livestock health management standards in 2021 required farms in wild boar habitats to install protective fences, limiting contact between farm pigs and wild boars. This measure is likely a key factor in the significant decrease in HEV incidence among pigs in Japan in recent years (25). This management approach could serve as a valuable model for reducing interactions between wild boars, livestock, and humans, thereby curbing the transmission of HEV.

Our study has several limitations. First, articles were sourced from only six databases, which may have excluded relevant literature from other databases. While we included studies from six major databases, our exclusion of studies published in non-English or non-Chinese languages could have led to a selection bias, potentially overlooking important regional findings. Second, the study focused on articles in English and Chinese, potentially overlooking studies published in other languages. Third, some of the studies had small sample sizes, which could have impacted the reliability of the overall estimates. Fourth, the risk factors we extracted, such as sample type and site, may not have been fully comprehensive, limiting our ability to analyze other factors that could influence anti-HEV IgG antibody positivity rates and HEV RNA detection. Fifth, despite including 87 eligible studies, research on swine HEV infection in certain regions of China remains insufficient. As such, future research should aim to better characterize and quantify these regional risk factors.

HEV seropositivity in pigs in China is notably high, with widespread infection across pig farms in all regions. Implementing biosecurity measures on pig farms may significantly contribute to the prevention and control of HEV, and tailored interventions in different regions could help reduce local infection rates. In conclusion, our findings underscore the importance of biosecurity measures in reducing HEV prevalence in pig populations. Given the potential for human transmission through pork products, enhanced surveillance programs at slaughterhouses, coupled with stricter biosecurity protocols on farms, are crucial in mitigating the public health risks associated with HEV. Further research should focus on understanding the effectiveness of region-specific interventions in controlling HEV transmission. Consequently, effective management and monitoring strategies are essential to mitigate HEV infection in pigs, thereby safeguarding public health. Moreover, pig slaughterhouses, as critical points of pork production, are also contaminated with HEV, presenting a potential risk to human health. Routine monitoring of slaughterhouses is crucial to assess the risk of HEV transmission to humans. Future research should focus on characterizing the genotype-specific transmission patterns of HEV and assess the long-term impact of biosecurity interventions. Additionally, studies evaluating the role of wild boars in HEV transmission to both pigs and humans are urgently needed to develop targeted control strategies. Therefore, comprehensive and ongoing surveillance programs should be established nationwide to gather more epidemiological data on swine HEV infections.

## Data Availability

The original contributions presented in the study are included in the article/supplementary material, further inquiries can be directed to the corresponding author.
